# Acute Intestinal Obstruction Due to Obturator Hernia: A Case Series

**DOI:** 10.7759/cureus.58901

**Published:** 2024-04-24

**Authors:** Althea V Cardoz, Ravi R Kumar, Niladri Banerjee, Shashi R Kumar

**Affiliations:** 1 General Surgery, All India Institute of Medical Sciences, Jodhpur, IND; 2 Surgical Disciplines, All India Institute of Medical Sciences, New Delhi, IND; 3 General Surgery, Nalanda Medical College and Hospital, Patna, IND

**Keywords:** obturator hernias, obturator foramen, anatomical repair, bowel gangrene, acute intestinal obstruction, obstructed obturator hernia

## Abstract

Most obturator hernias are diagnosed intraoperatively due to their vague signs and symptoms. However, they are associated with a high mortality rate mainly because of the patient's age, comorbidities, and late diagnosis. We present three cases of obturator hernia in patients admitted under our care with signs of acute intestinal obstruction. All the patients were elderly with comorbidities, and they underwent open surgery with anatomical repair of the hernial defect with or without resection of any gangrenous bowel. They were discharged in good health, and during the limited follow-up period, there has been no recurrence. We would like to emphasize that obturator hernia should be considered in the differential diagnosis when an elderly, thinly built woman presents with acute intestinal obstruction. Though the outcome of such cases depends on the clinical status and comorbidities of the patient, early diagnosis and treatment can help in reducing postoperative morbidity and mortality.

## Introduction

Of all abdominal wall hernias, the incidence of obturator hernia is 0.07-1.6% [1]. Since this hernia is usually not picked up by inspection or palpation, it remains undiagnosed clinically. Small bowel obstruction occurs in approximately 0.2-1.6% of all cases, predominantly in the right-sided hernias [2]. More than 60% of these cases are diagnosed at laparotomy/laparoscopy due to the nonspecific signs and symptoms [3]. The reported postoperative morbidity and mortality rates range from 17.6% to 86% and from 0% to 47.6%, respectively, which have been attributed to the delay in diagnosis and surgical treatment rather than the techniques of repair [4]. We present here a series of three cases of obturator hernia presenting as an acute intestinal obstruction that were managed surgically. This is an attempt to reiterate the fact that prompt diagnosis and management of this condition will lead to good postoperative recovery, irrespective of the age and comorbid status of the patients.

## Case presentation

Case one

A 70-year-old lady presented to the emergency department (ED) with signs of intestinal obstruction. She had a similar episode one year back for which she was managed conservatively. She was diabetic, on insulin, with no surgical history. General examination was within normal limits with no fever, tachycardia, or hypotension. She had a distended, non-tender abdomen, without any obvious swelling or cough impulse externally at the hernial sites. Per rectal examination was done, and the rectum was empty. Laboratory findings were not suggestive of any sepsis, liver or renal failure, or any electrolyte disturbance. However, her blood sugars were grossly deranged, for which she was started on an insulin infusion. A CT scan showed a left-sided obturator hernia.

**Figure 1:**
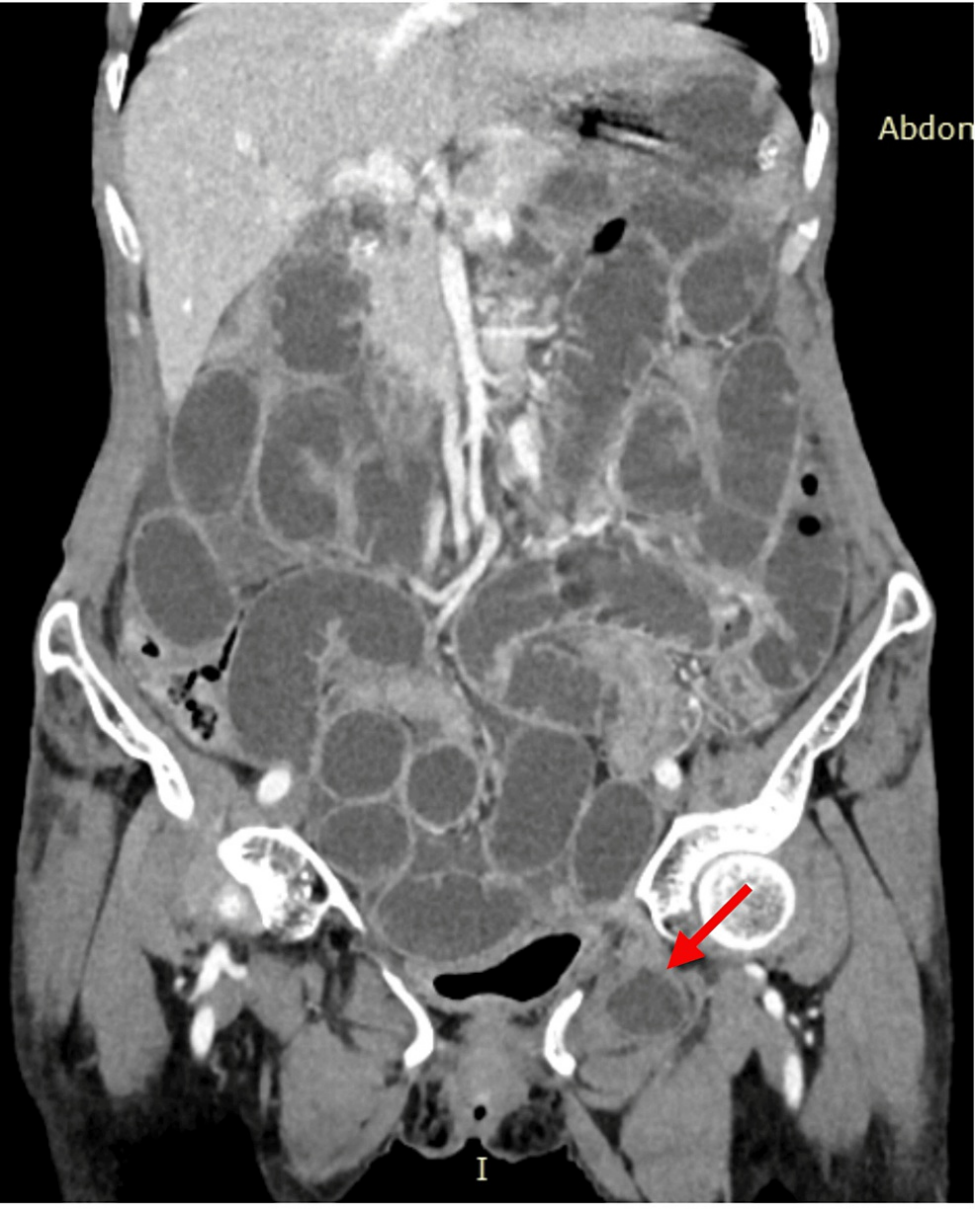
CT scan showing left-sided obturator hernia Arrow indicates the herniated small bowel loop through the obturator foramen

Treatment

**Figure 2:**
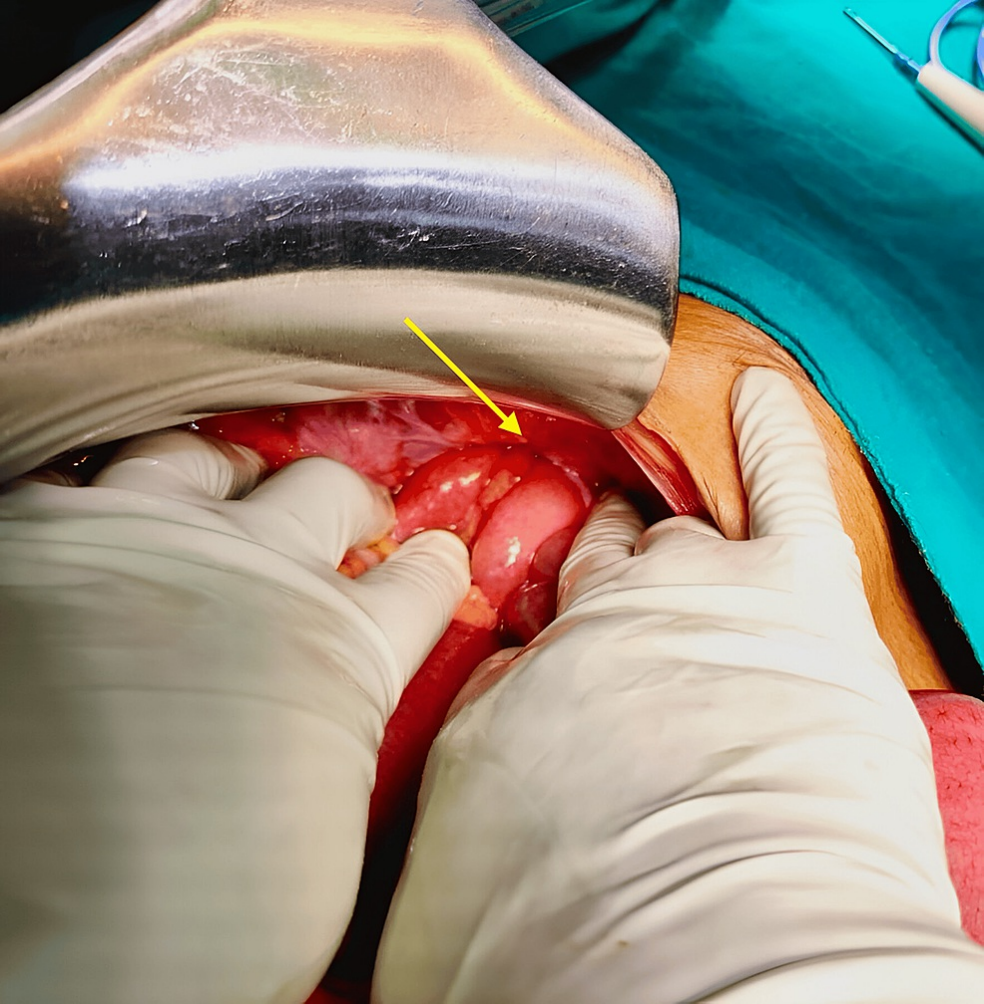
Left side obturator hernia Arrow indicates the herniated small bowel loop through the obturator foramen

**Figure 3:**
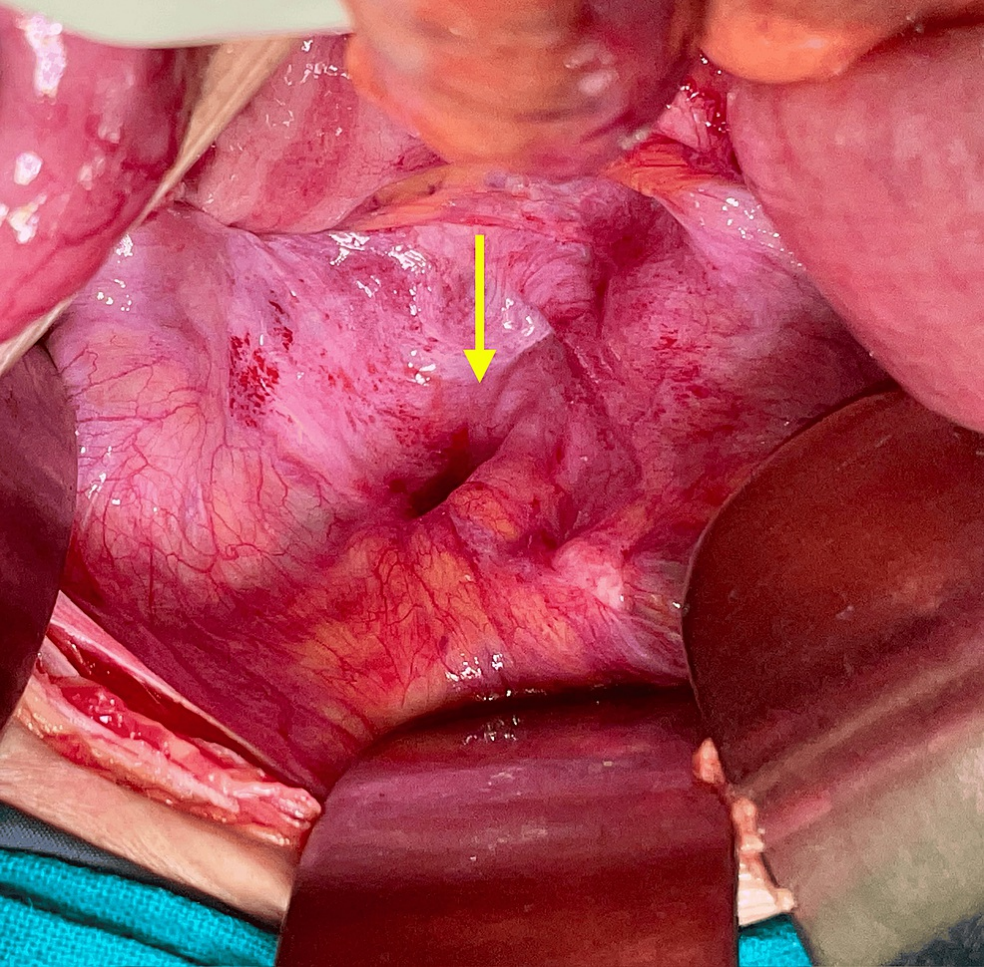
Widened obturator foramen Obturator foramen is seen after the reduction of the small bowel loop (arrow)

In view of the comorbidity, the patient was admitted to the surgical ICU, and an emergent laparotomy was performed. Intraoperatively, a loop of the ileum was seen herniating through the left obturator canal, which was gently reduced (Figure 2,3). There was congestion in the entrapped bowel loop, which gradually returned to its normal color post-reduction. The obturator defect was closed by anatomical closure with polypropylene sutures. The postoperative period was uneventful, and she was discharged on postoperative day five. 

Outcome and Follow-up

The lady recovered well postoperatively, with no recurrence within two years of follow-up.

Case two

**Figure 4:**
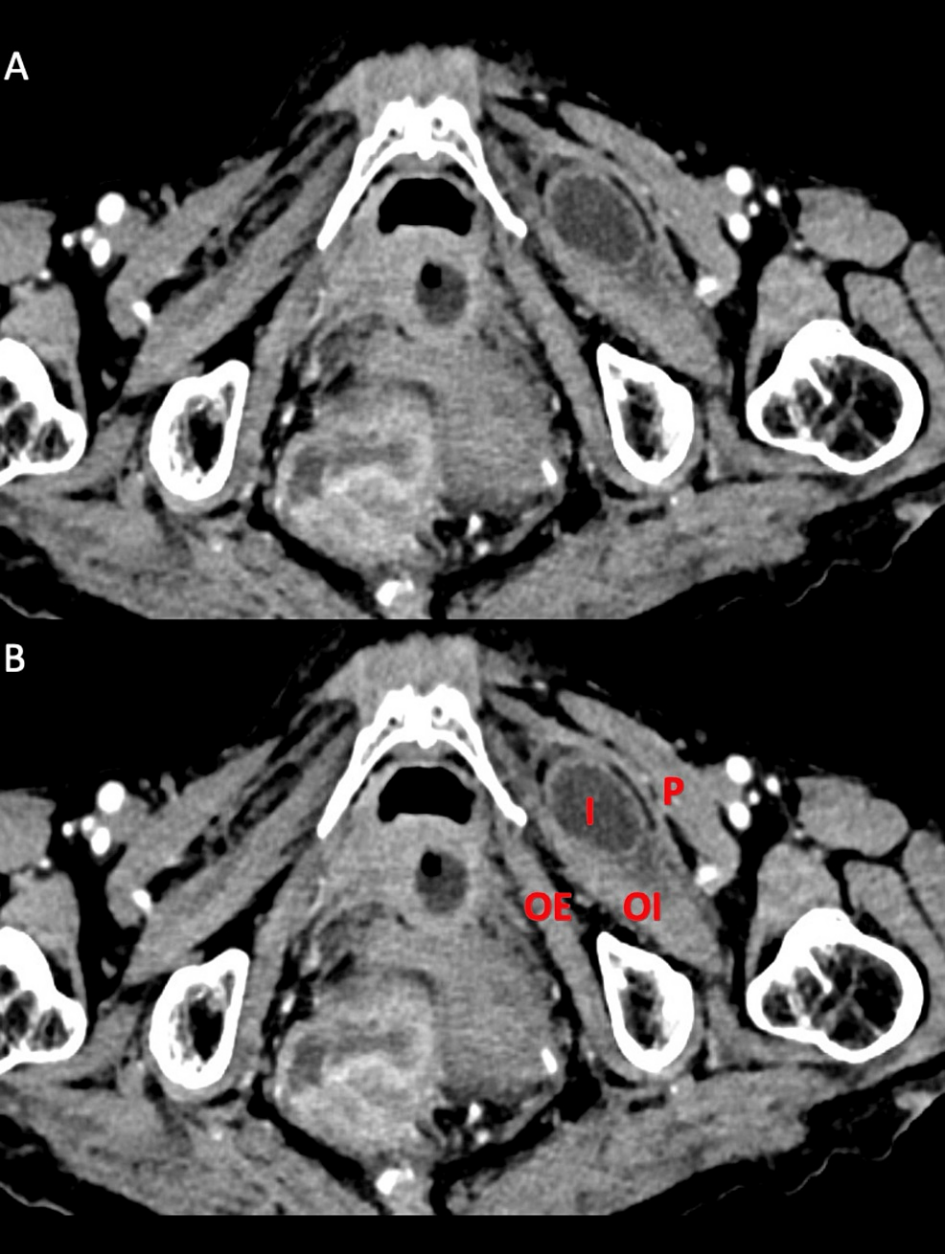
Contrast-enhanced CT showing obturator hernia with bowel as content Contrast-enhanced CT images of the abdomen in a case of intestinal obstruction. Axial (A-unmarked, B-marked) images show the left-sided obturator hernia with a dilated ileal loop (I) lying in between the obturator internus (OI) and pectineus (P) muscles. OE - obturator externus muscle

A 70-year-old gentleman presented to the ED with complaints of abdominal pain, vomiting, and obstipation for two days. On examination, he had tachycardia (pulse rate of 106/min) with a distended and diffusely tender abdomen. His per rectal examination was normal. Investigations revealed hypoalbuminemia and hypokalemia along with a deranged coagulation profile (INR of 2.78). Contrast-enhanced CT showed obturator hernia with bowel as content (Figure 4).

Treatment

**Figure 5:**
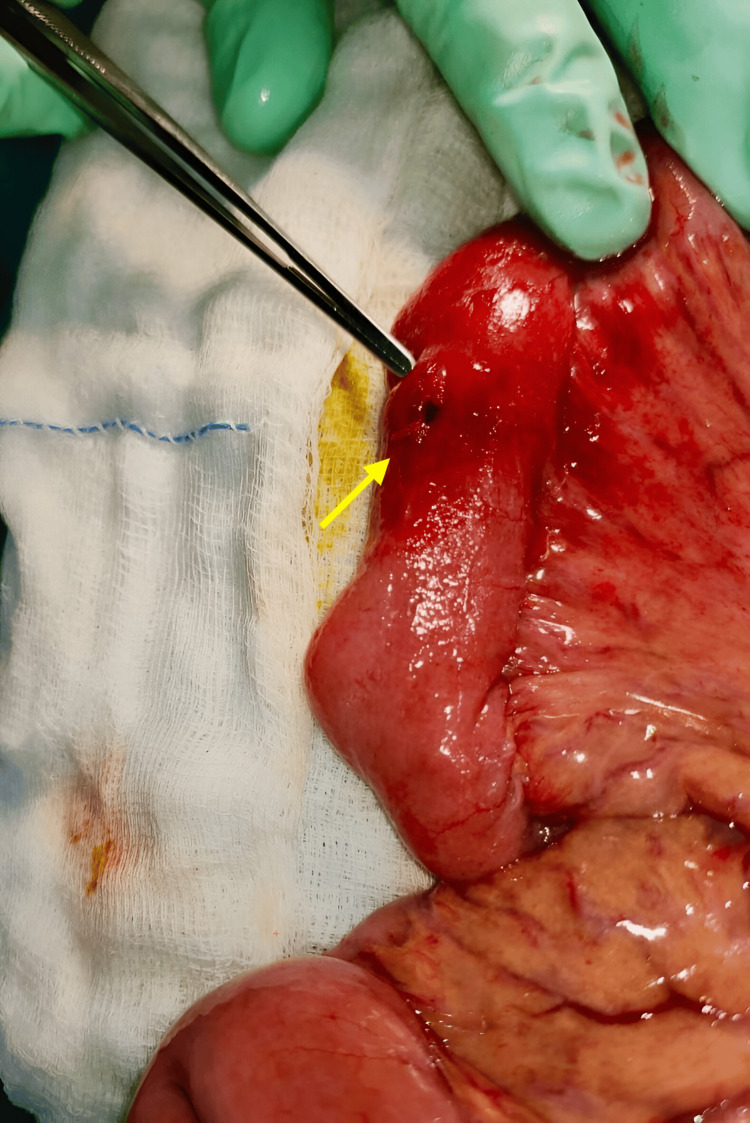
Reduced bowel loop with perforation Arrow indicates the perforation in the small bowel, seen after reduction of the content (small bowel)

**Figure 6:**
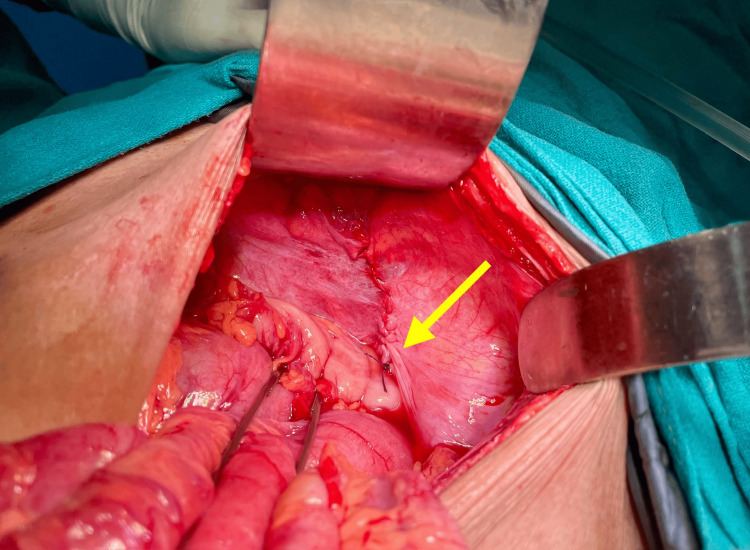
Post-anatomical repair of the defect Arrow indicates the closed hernial defect by anatomical repair without mesh

The gentleman was optimized and then taken up for an exploratory laparotomy through a midline infraumbilical incision. The hernia was identified, with a perforated gangrenous small bowel loop as the content (Figure 5). The involved bowel loop was resected, and the two ends were brought out as a double-barrel ileostomy (Figure 6). The hernial defect was closed anatomically with polypropylene sutures. The abdominal cavity was thoroughly washed, and a mesh laparostomy was performed according to damage control principles. In the postoperative period, the patient was on ventilatory support for five days due to his comorbidities and general nutritional status, but he gradually improved and was discharged from the hospital on postoperative day 10.

Outcome and Follow-up

The gentleman recovered well postoperatively, has undergone reversal of stoma, and has had no recurrence in 14 months of follow-up.

Case three

**Figure 7:**
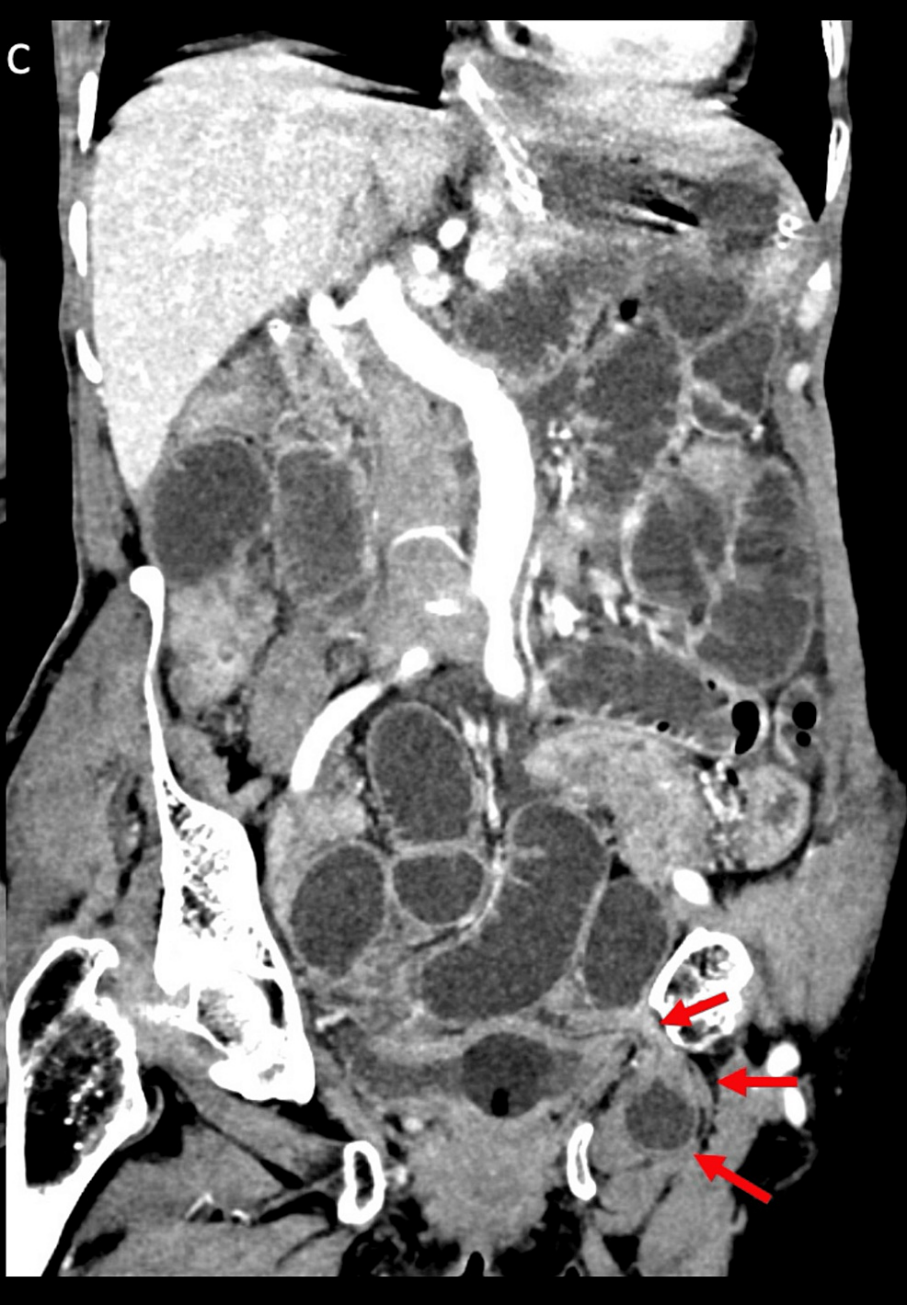
Contrast-enhanced CT showed obturator hernia in oblique coronal view Oblique coronal image showing the whole course of the obturator hernial sac (red arrows) along with proximally dilated jejunal and ileal loop.

A 90-year-old lady presented with complaints of recurrent vomiting, abdominal distension, and abdominal pain for five days. On examination, she was a thin-built, frail lady with a pulse of 95 per minute and a distended, non-tender abdomen with no palpable lump or cough impulse. Her per rectal and per vaginal examinations were normal. She was a known hypertensive on irregular medications and had deranged liver functions. Contrast-enhanced CT showed an obturator hernia (Figure 7).

Treatment

**Figure 8:**
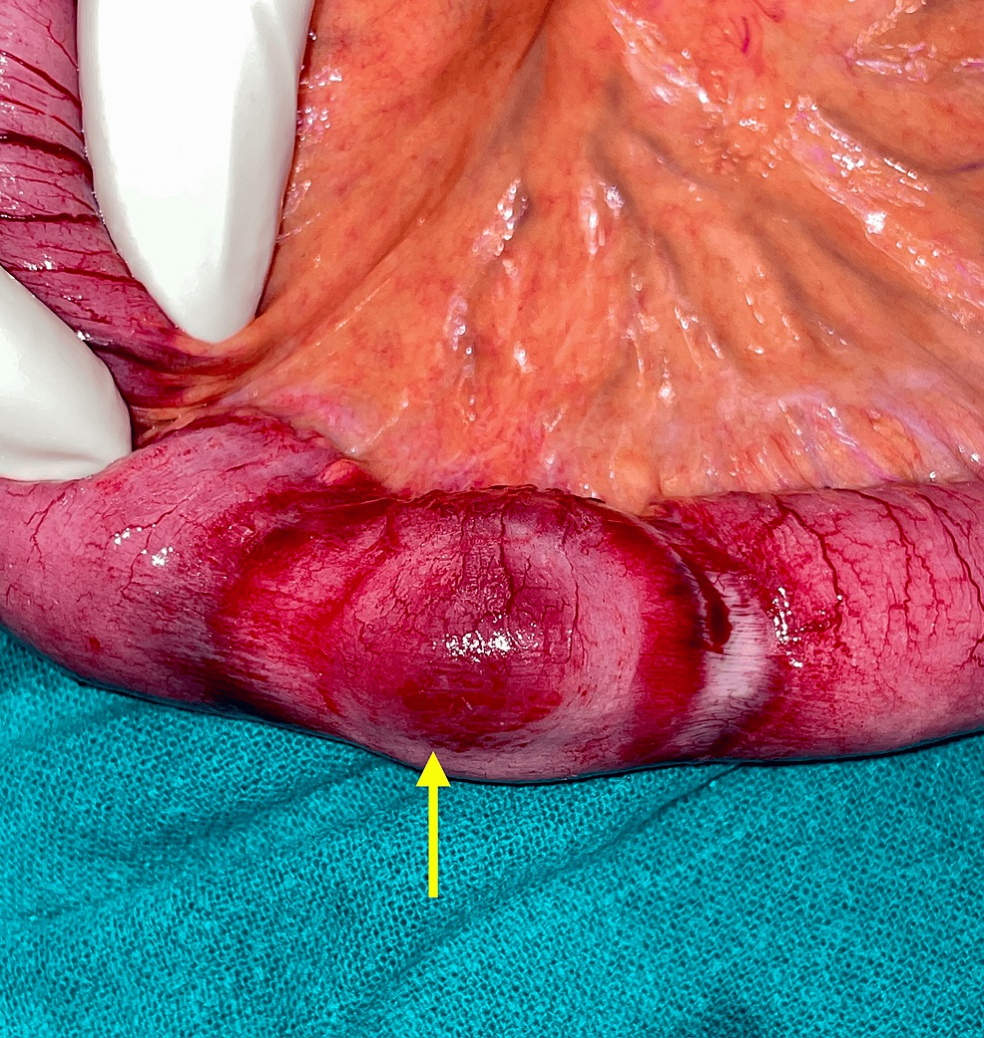
Ischemic bowel loop seen after reduction of herniated loop Arrow indicates the ischemic bowel loop

After optimization, the lady underwent an exploratory laparotomy, which revealed the left-sided obturator hernia, with a small bowel as the content. After reduction of the hernial contents, the small bowel loop was found to be dusky and non-viable (Figure 8). Hence, a resection of the involved segment along with a side-to-side bowel anastomosis was performed. The hernial defect was anatomically repaired with polypropylene sutures, the abdominal cavity was thoroughly washed, and closed over a drain. The postoperative period was uneventful, and she was discharged on postoperative day seven.

Outcome and Follow-up

The lady recovered well postoperatively, with no recurrence within 11 months of follow-up.

## Discussion

In 1724, Armaud de Ronsil was the first to describe obturator hernia, and Henry Obre was the first to successfully treat it in 1851 [5]. An obturator hernia is an uncommon type of pelvic hernia, commonly seen in thinly built, elderly females (also called "little old lady's hernia"). This is due to their wide pelvis, the greater transverse diameter, and a more triangular obturator canal [6]. Two of our patients were elderly females, whereas one was a 70-year-old gentleman.

An obturator hernia is more commonly seen on the right side, with a six percent incidence of bilaterally. It has been linked to the fact that the presence of the sigmoid colon on the left side covers the obturator canal [7]. However, all three of our patients presented with left-sided hernias.

The signs and symptoms of obturator hernias are vague, and it does not commonly present with acute intestinal obstruction. Pain and paresthesia on the medial aspect of the thigh up to the knee joint with the inability to adduct the thigh are seen in 15-50% of the cases. This occurs due to the compression of the obturator nerve by the hernial sac and is known as the Howship-Romberg sign [8]. Another sign, the Hannington-Kiff sign, which is the absence of the adductor reflex of the ipsilateral thigh, may also be seen in a few cases [5]. However, none of these signs were seen in our patients.

Radiological investigations in the form of abdominal and pelvic ultrasonography and computed tomography (CT) are key to making an early diagnosis. Due to the deep location of the hernia, the diagnosis of hernia by ultrasound may be difficult [9]. A contrast-enhanced CT scan is the best tool to accurately identify the presence of an obturator hernia. It can also determine the vascularity of the herniated bowel and confirm the presence of a similar hernia on the opposite side. All our patients underwent a contrast-enhanced CT scan, which confirmed the diagnosis.

The management of obturator hernia is by surgery, which is required in all cases. The various surgical approaches include lower midline laparotomy, retroperitoneal approach, obturator or crural approach, inguinal approach, laparoscopy, and combined. The lower midline approach is the most used approach, especially in cases where the diagnosis is not available at the time of the surgery, because it helps in exploring bilaterally. Laparoscopy has the advantage of being minimally invasive, identifying the presence of other hernias, lower post-operative morbidity, and early discharge [2]. However, the general condition of the patient, as well as the condition of the bowel (strangulation/gross dilatation), may be unfavorable for a laparoscopic approach, and in those cases, the open approach is used. Two of our patients had uncontrolled medical comorbidities, and the other patient had a perforated gangrenous bowel in the hernial sac; thus, all of them underwent open surgery.

The most common content of the hernial sac is the small bowel. More commonly, it is a Richter type of hernia. Other contents of the hernial sac can be the appendix, omentum, urinary bladder, large bowel, uterus, ovaries, and fallopian tubes. The repair of the hernial defect can be done either by placing a mesh or by anatomical approximation of surrounding tissues with polypropylene sutures. We chose to do an anatomical repair in our patients, and in our limited follow-up period, we have not come across any recurrence.

## Conclusions

Obturator hernia presents a diagnostic challenge due to its vague symptoms and deep location. While it is more commonly seen in elderly females, it can also affect males. Despite being more prevalent on the right side, all our cases were on the left side. Although classical signs like Howship-Romberg and Hannington-Kiff were absent in our patients, radiological imaging, particularly contrast-enhanced CT scans, played a crucial role in confirming the diagnosis. Surgical intervention remains the cornerstone of treatment. Laparoscopy can be considered if the patient is hemodynamically stable and the proximal bowel is not significantly dilated. In our cases, open surgery was necessitated by patient factors and bowel condition. Anatomical repair with polypropylene sutures was chosen, showing promising results with no reported recurrence during the follow-up period. Overall, a comprehensive understanding of obturator hernia presentation, diagnostic modalities, and surgical management is essential for successful outcomes.
